# Genome-wide identification and functional characterization of oleosin genes in peanut (*Arachis hypogaea* L.)

**DOI:** 10.3389/fpls.2025.1623513

**Published:** 2025-08-06

**Authors:** Meiling Hu, Jie Wu, Xiaomeng Xue, Li Huang, Nian Liu, Liying Yan, Yuning Chen, Xin Wang, Yanping Kang, Zhihui Wang, Huifang Jiang, Boshou Liao, Yong Lei, Dongxin Huai

**Affiliations:** Key Laboratory of Biology and Genetic Improvement of Oil Crops, Ministry of Agriculture and Rural Affairs, Oil Crops Research Institute of Chinese Academy of Agricultural Sciences, Wuhan, China

**Keywords:** peanut, oil-body-membrane protein, oleosin, fatty acid, oil content

## Abstract

Peanut is a worldwide important oil crop and serves as a major source of vegetable oil. Seed oil is stored as oil bodies (OB), which are subcellular structures in the cytoplasm. Oil bodies accumulate triacylglycerols (TAGs) inside and surrounded by a monolayer of phospholipids (PL) with oil-body-membrane proteins. Oleosins have been demonstrate to be the predominant oil-body-membrane proteins and played a crucial role in maintaining oil body stability. In this study, 12 oleosin genes were identified in peanut, distributed across 9 chromosomes and classified into three lineages (U, SH, and SL). Most *AhOle* genes exhibited high expression levels in developing seeds, a pattern that aligns with the expression profiles of U, SH, and SL oleosins. Several cis-elements were found in the promoters of *AhOle* genes, such as LTR, ABRE, and TCA-element. Expression analysis confirmed that these genes were responsive to treatments involving drought, cold stress and various plant hormones. The *AhOle11* gene was cloned due to its highest expression level observed during seed development. Subcellular localization analysis demonstrated that *AhOle11* gene was localized in oil bodies. Overexpression *AhOle11* in *Arabidopsis* significantly increased in seed oil content and an increased oil body density, thereby supporting its critical role in oil accumulation. Nevertheless, the fatty acid profiles in transgenic seeds exhibited only minor alterations. This study contributes to a comprehensive understanding of the oleosin family in peanut and provides valuable insights for genetic improvement aimed at enhancing oil accumulation.

## 1 Introduction

Peanut (*Arachis hypogaea* L.) is one of the important oilseed crop worldwide, which are rich in oil, protein, sugar, resveratrol and other nutrients (Bishi et al., 2015). The peanut kernel contains 45-58% oil, primarily composed of palmitic (C16:0), steric (C18:0), oleic (C18:1), linoleic (C18:2), arachidic (C20:0), eicosenoic (C20:1), behenic (C22:0) and lignoceric (C24:0) acids (Chapman and Ohlrogge, 2012; Shasidhar et al., 2017). Seed oil is stored as triacylglycerol (TAG) within oil bodies (OB), where they serve as a primary source of energy and nutrients during germination (Chapman and Ohlrogge, 2012). The unique structure of oil bodies allows for efficient storage and mobilization of TAGs, which are the most energy-dense form of lipids.

Oil bodies are composed of a central core of TAGs encased by a monolayer of phospholipids (PL), which is embedded with oil-body-membrane proteins such as oleosin, caleosin, and steroleosin (Hsieh and Huang, 2004; Manan et al., 2017; Shao et al., 2019). Oleosins are the most abundant and well-studied proteins in oil bodies. They are characterized by a long hydrophobic hairpin structure that spans the monolayer, with a hydrophilic N-terminal domain exposed to the cytoplasm (Tzen and Huang, 1992; Frandsen et al., 2001; Shao et al., 2019; Zhao et al., 2022). Oleosins, with their unique structure and abundance, are primarily involved in stabilizing the oil body and preventing its fusion with other oil bodies (Huang, 1994; Deleu et al., 2010; Huang and Huang, 2015; Shimada and Hara-Nishimura, 2015). The oleosin genes have been identified across a diverse range of organisms, including algae, moss and higher plants (Huang, 1996). Based on their amino acid sequences and tissue-specific expression patterns, oleosin genes are divided into six lineages: primitive (P oleosin), universal (U oleosin), seed low-molecular-weight (SL oleosin), seed high-molecular-weight (SH oleosin), tapetum oleosins (T oleosin), and mesocarp (M oleosin) (Fang et al., 2014; Huang and Huang, 2015). The P lineage is the most ancestral and is found in green algae, mosses, and ferns (Huang and Huang, 2015, 2016). The U lineage is universally present in all land plants and is characterized by a conserved C-terminal AAPGA motif (Zou et al., 2024). The SL and SH lineages are seed-specific, with the SL clade evolving first from the U clade and later giving rise to the SH, M, and T lineages (Shimada et al., 2008). The M lineage is present in *Lauraceae*, while the T lineage has been exclusively detected in the tapetum of *Brassicaceae* (Chen et al., 2019).

Oleosins play several important roles in plant cells. One of their primary functions is to stabilize oil bodies, preventing their aggregation and fusion. The absence of oleosins leads to the compression and fusion of oil bodies, resulting in enlarged oil bodies within *AtOLE1*-knockout *Arabidopsis* seeds (Shimada et al., 2008). Additionally, oleosins are involved in lipid metabolism, influencing the oil content and fatty acid composition (Siloto et al., 2006; Hu et al., 2009; Liu et al., 2013; Hu et al., 2023). Down-regulating the expression levels of oleosins in *Arabidopsis* resulted in a significantly reduction in oil content (Shimada et al., 2008; Wu et al., 2010). Over-expression oleosin genes in *Arabidopsis* led to altered fatty acid composition and a slight increase in oil content (Chen et al., 2019; Ojha et al., 2021). Oleosins also contribute to the stress response by modulating oil body size and number in accordance with metabolic demands. The analysis of the expression profiles of *Theaceae* oleosin genes revealed that SL2 oleosin was up-regulated, whereas SL1 and SL3 oleosins were down-regulated under drought stress (Zhang et al., 2023). Furthermore, SH1 and SH4 oleosins were up-regulated in response to both cold and heat stress (Zhang et al., 2023). When the *Atole1* mutant was exposed to freezing conditions, its germination rate decreased by approximately 50% (Shimada et al., 2008). However, the overexpression of *SbOle1*, *SbOle2*, and *SbOle3* in *Atole1* mutant significantly restored normal germination rates under freezing conditions (Ojha et al., 2021).

Oleosins are crucial for lipid storage, metabolism, and oil body dynamics; however, the characteristics and functions of oleosins in peanut remain largely unknown. In this study, oleosin genes from the ShiTouQi cultivar were identified, and their physical and chemical properties, gene structure, phylogenetic tree, and expression pattern were analyzed. One of them with the highest expression level in developing seeds was cloned and its function was characterized in *Arabidopsis*. This study aims to establish a foundation for an understanding of molecular biological functions of peanut oleosin genes and provides valuable insights for genetic improvement efforts focused on enhancing oil content in peanut.

## 2 Materials and methods

### 2.1 Identification of peanut oleosin genes members and properties analysis

The genomic sequences of a cultivated peanut *A. hypogaea* cv. ShiTouQi were downloaded from Peanut Genome Resouce (http://peanutgr.fafu.edu.cn/index.php). The peanut oleosin genes were identified using two tools: BLASTP search and HMMER. The protein sequences of *Arabidopsis thaliana* oleosin genes, obtained from TAIR (https://www.arabidopsis.org/), were used as queries for BLASTP searches against the annotated peanut proteins and to identify peanut oleosin homologs. The conserved oleosin domain (PF01277) was then employed to identify the proteins through HMMER program (Finn et al., 2011). By integrating the sequences derived from both methods, peanut oleosin proteins were identified.

The gene structure information was retrieved from Peanut Genome Resouce (http://peanutgr.fafu.edu.cn/index.php). The chromosome location, gene structure and conserved domain were visualized using MG2C (https://qiaoyundeng.github.io/) and TBtools (Chao et al., 2021). The predicted molecular weights and theoretical isoelectric points (PI) were calculated using Expasy - ProtParam (https://web.expasy.org/protparam/). The conserved domains were identified using Pfam (http://pfam.xfam.org/).

### 2.2 Gene duplication and phylogenetic analysis of peanut oleosin genes

The oleosin protein sequences of rapeseed (*Brassica napus* L.) were retrieved from BnTIR (https://yanglab.hzau.edu.cn/BnTIR). The oleosin protein sequences of *Arachis duranensis* and *Arachis ipaensis* were obtained from Peanut Base (https://www.peanutbase.org/). Multiple sequence alignments of the oleosin protein sequences were performed using ClustalW software (Higgins and Sharp, 1988). A phylogenetic tree was constructed using the neighbor-joining method in the MEGA X, employing 1000 bootstrap replicates (Kumar et al., 2018). The evolutionary tree was visualized using the online tool iTOL (https://itol.embl.de/itol.cgi).

The collinearity within and between *A. hypogaea*, *A. duranensis* and *A. ipaensis* was established using MCScanX (http://chibba.pgml.uga.edu/duplication/) incorporated into TBtools. The collinear relationships of oleisin genes were drawn using Circos (Krzywinski et al., 2009).

### 2.3 Cis-elements in the promoter regions of peanut oleosin genes

The promoter sequences of peanut oleosin genes were extracted from the ShiTouQi genome using TBtools, encompassing a 2000bp DNA sequence upstream of the ATG start codon. The cis-elements in the promoter regions were predicted using New PLACE websites (https://www.dna.affrc.go.jp/PLACE/?action=newplace). The positions of cis-elements were visualized using TBtools.

### 2.4 Expression profile analysis of peanut oleosin genes

The multi-tissue transcriptome data used in this study were downloaded from Peanut Genome Resouce (http://peanutgr.fafu.edu.cn/index.php). Additionally, transcriptome data of leaves subjected to drought and cold stress, as well as those treated with plant hormone, were also obtained from this database. The heat maps were generated using TBtools.

### 2.5 RNA extraction and RT-PCR

To validate the expression profile, roots, leaves, stems, flowers, and developing seeds at 20, 30, 40, 50, and 60 days after pollination (DAP) of Zhonghua 12 were collected and five development stages as described previously (Huai et al., 2020). Total RNA was extracted using RNAprep Pure Plant Kit (DP 441, TIANGEN, China). First strand cDNA was syntheisized from 1 µg RNA using HiScript III 1st Strand cDNA Synthesis Kit (R 312-02, Vazyme). Quantitative real-time PCR (RT-qPCR) was performed according to the instructions of ChamQ Universal SYBR qPCR Master Mix (Vazyme, China). The primers for RT-qPCR were designed using NCBI (**Supplementary Table S3**). The relative expression levels of genes were calculated by the 2^−△△CT^ method. The experiment was performed using three biological replicates.

### 2.6 Cloning of *AhOle11*

Total RNA was extracted from developing seeds of Zhonghua 12 at 40 days after pollination using TRIzol reagent (DP 424, TIANGEN, China), following the manufacturer’s protocol. Reverse transcription was performed using HiScript IV 1st Strand cDNA Synthesis Kit (R412-01, Vazyme, China) as described by the manufacturer. The *AhOle11* (*AH16G32210*) gene was cloned using the primers: 5’-ATGTCTGATCAAACAAGGACA-3’ and 5’- TCAATACCCTTGTGTGCCCTC-3’.

### 2.7 Subcellular localization of *AhOle11*

The *AhOle11* gene without a stop codon were amplified by PCR, and inserted between the cauliflower mosaic virus (CaMV) 35S promoter and the green fluorescent protein (GFP) gene. The resulting plasmids were designated as pHBT-AhOle11-GFP. The empty vector pHBT-GFP was analyzed as a control. The *AtOle5* (*AT3G27660*) from *Arabidopsis* was fused with the red fluorescent protein (RFP) gene. The p35S:AtOle5-RFP construct was used as an oil body marker. The pHBT-GFP and pHBT-AhOle11-GFP were each transiently co-expressed with the oil body marker in *Arabidopsis* protoplasts by PEG transformation, respectively (Yoo et al., 2007). Fluorescence signals were detected using a confocal laser scanning microscope (Nikon C2-ER, Japan). GFP was excited at 488nm, while RFP was excited at 561 nm.

### 2.8 Expression of *AhOle11* in *Arabidopsis*

The *AhOle11* gene was amplified via PCR and inserted into the pBinGlyRed2 vector, which contains a DsRed2 driven by 35S promoter. As a result, the *AhOle11* was positioned between the seed-specific soybean glycinin-1 promoter and its 3’UTR.The recombinant plasmid was introduced into *Agrobacterium tumefaciens* strain GV3101 and transformed into *Arabidopsis* by the floral dip method. DsRed-positive seeds were identified using a green LED flashlight with a red camera filter lens (Huai et al., 2023).

### 2.9 Analysis of seed oil content and fatty acid composition

The oil content and fatty acid composition of mature DsRed-positive *Arabidopsis* seeds were analyzed as previously described (Huai et al., 2018). In brief, 20 mg grounded seeds were placed into a glass tube. Subsequently, 1.5ml of 2.5% sulfuric acid-methanol solution, 0.35 ml of toluene and 0.3 ml of C15:0 (internal standard) solution in methanol (10 mg/ml) were added. Fatty acids were transmethylated at 90°C for 30–45 min. After cooling to room temperature, 1 ml H_2_O and 1 ml hexane were added into the tube. The supernatant was filtered through a 0.45 μm microporous membrane and transferred into an autosampler vial. The fatty acid contents were determined by gas chromatograph (GC) using an Agilent 7890B with flame ionization detection and the DB-23 column. The concentration of fatty acids methyl esters (FAMEs) was quantified based on the ratio of different FAMEs peak areas relative to the internal standardization (C15:0). The fatty acid composition is expressed as absolute concentrations (mg/g). Three biological replicates per line were analyzed in this experiment. Statistically significant differences were analyzed using Student’s t-test by the SPSS SPSS19.0; The phenotypes was performed on GraphPad Prism 8.0.

### 2.10 Visualization of oil body

Fixation of seeds and lipid staining were performed as previously described with modifications (Cai et al., 2015; Zhang et al., 2019). Mature seeds were imbibed in distilled water for 20~30 min for seed coat removal. Then embryos were immediately immersed in formalin-acetic acid-alcohol (FAA) fixative (formalin: acetic acid: 50% ethanol = 1:1:18 v/v) for at least 24 h. The fixed tissues were trimmed using a scalpel and subsequently immersed in 15% sucrose solution at 4°C until they sank. They were then transferred to a 30% sucrose solution at 4°C and incubated until they sedimented. The fixed tissues were embedded with optimal cutting temperature compound (OCT) compound. Cryosections (8~10 µm) were prepared using a Cryostat Microtome (Thermo, CRYOSTAR NX50, USA). The cryosections were stained with BODIPY 493/503 for 20 min and subsequently washed with PBS (pH 7.4). Thereafter, the sections were re-stained with 4’,6-diamidino-2-phenylindole (DAPI) for 10 min in the dark, followed by additional washes with PBS (pH 7.4). Image observation for BODIPY and DAPI was performed using a confocal laser scanning microscope (Nikon C2-ER, Japan). DAPI was excited at 330-380nm, while BODIPY was excited at 488 nm.

## 3 Result

### 3.1 Identification of oleosin genes in peanut

**Figure 1:**
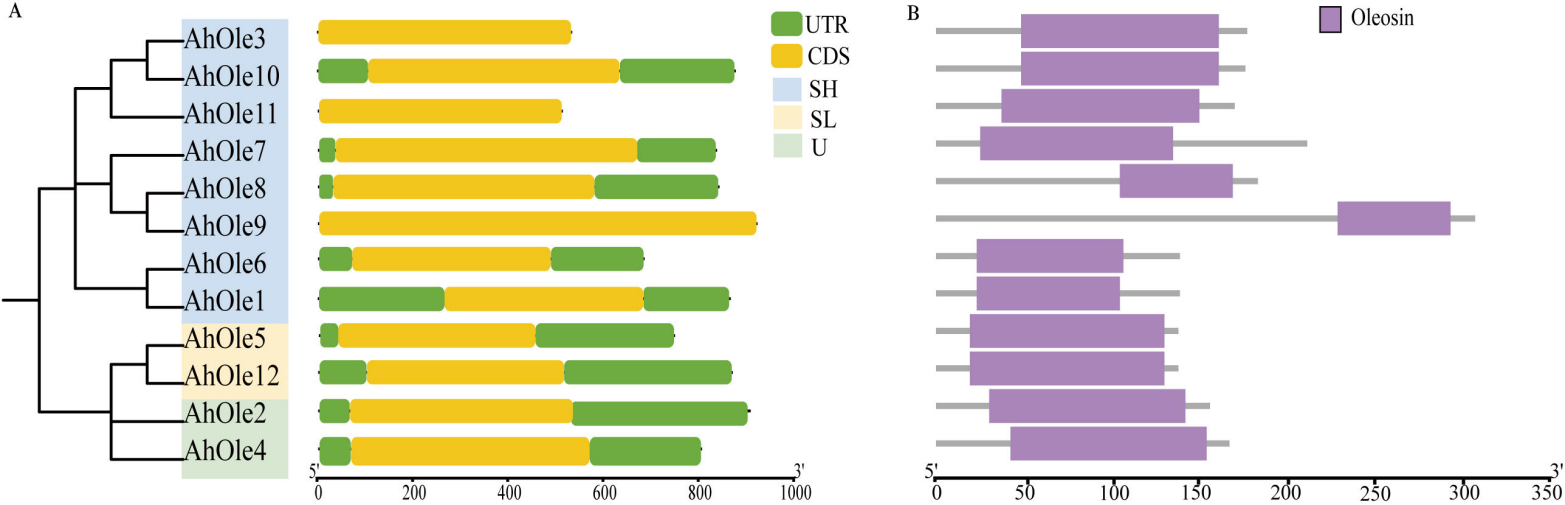
Gene structures and conserved domain of *AhOle* genes in Peanut. **(A)** Gene structure of *AhOle* genes. **(B)** The conserved domains of AhOles.

A total of 12 *oleosin* genes were identified in the peanut genome and were designated as *AhOle1*~*AhOle12* based on their chromosomal locations (**Table 1**). The coding sequences (CDSs) of peanut *oleosin* genes ranged from 414 to 918 bp in length, encoding proteins consisting of 137 to 305 amino acids, with molecular weights from 14.3 to 31.7 kDa (**Table 1**). All *AhOle* genes were intronless throughout their entire open reading frames (**Figure 1A**). Furthermore, all AhOles contained solely the oleosin domain (**Figure 1B**).

**Table 1:** Physicochemical properties of *oleosin* genes in peanut.

Gene name	Accession no.	Chr	Gene length	CDS	AA	MW (kDa)	PI	Chromosomal localization
AhOle1	AH01G03570	01	862	417	138	14.7	9.86	4486434~4487295
AhOle2	AH01G32400	01	903	468	155	17.1	9.46	107473444~107474346
AhOle3	AH05G24070	05	531	531	176	18.4	9.57	91311923~91312453
AhOle4	AH08G08850	08	801	501	166	16.9	8.99	16278126~16278926
AhOle5	AH10G26080	10	743	414	137	14.4	9.99	108045389~108046131
AhOle6	AH11G05190	11	681	417	138	14.6	9.99	6837323~6838003
AhOle7	AH11G20340	11	833	633	210	22.0	6.06	100900430~100901262
AhOle8	AH14G37650	14	838	549	182	19.4	4.70	127973098~127973935
AhOle9	AH14G37660	14	918	918	305	31.7	4.02	127981879~127982796
AhOle10	AH15G31410	15	874	528	175	18.4	9.57	145678796~145679669
AhOle11	AH16G32210	16	510	510	169	17.7	9.61	133246746~133247255
AhOle12	AH20G33740	20	865	414	137	14.3	10.08	138129036~138129900

**Figure 2:**
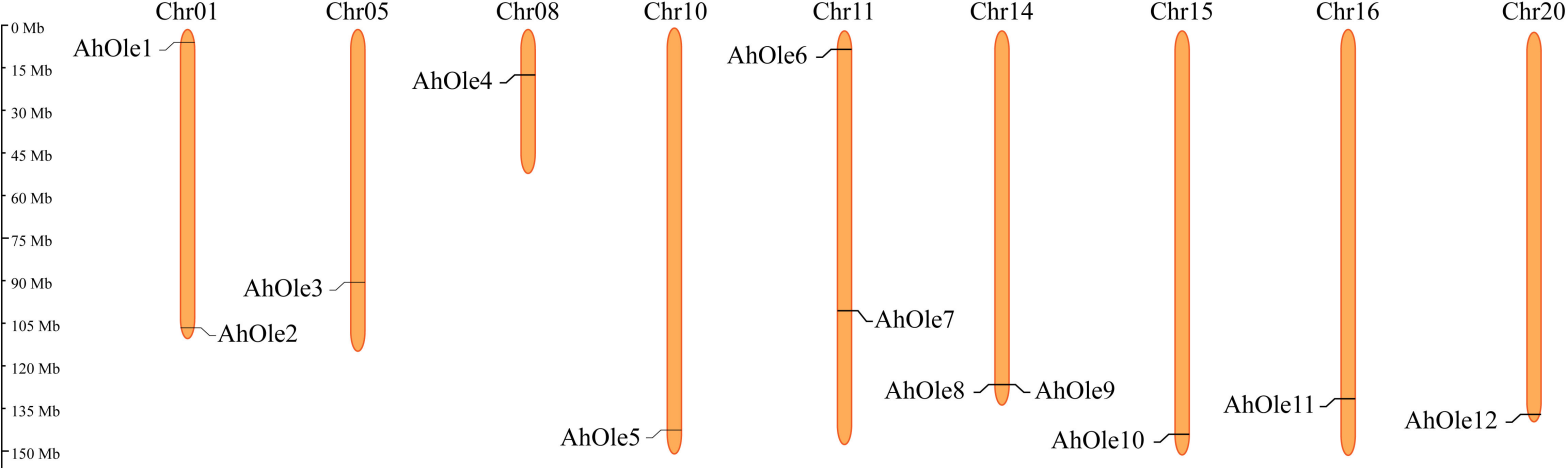
Chromosomes localization of the *oleosin* gene families in peanut.

The *AhOle* genes were unevenly distributed across 9 chromosomes in peanut, an allotetraploid comprising A and B subgenomes. Five *AhOle* genes was detected on Chr01, Chr05, Chr08 and Chr10 in subgenome A, whereas seven *AhOle* genes was located on Chr11, Chr14 Chr15, Chr16 and Chr20 in subgenome B. Notably, Chr05, Chr08, Chr10, Chr15, Chr16 and Chr20 each contained only a single *AhOle* gene, whereas Chr01, Chr11 and Chr14 each harbored two such genes (**Figure 2**).

### 3.2 Phylogenetic and synteny analysis of AhOles

**Figure 3:**
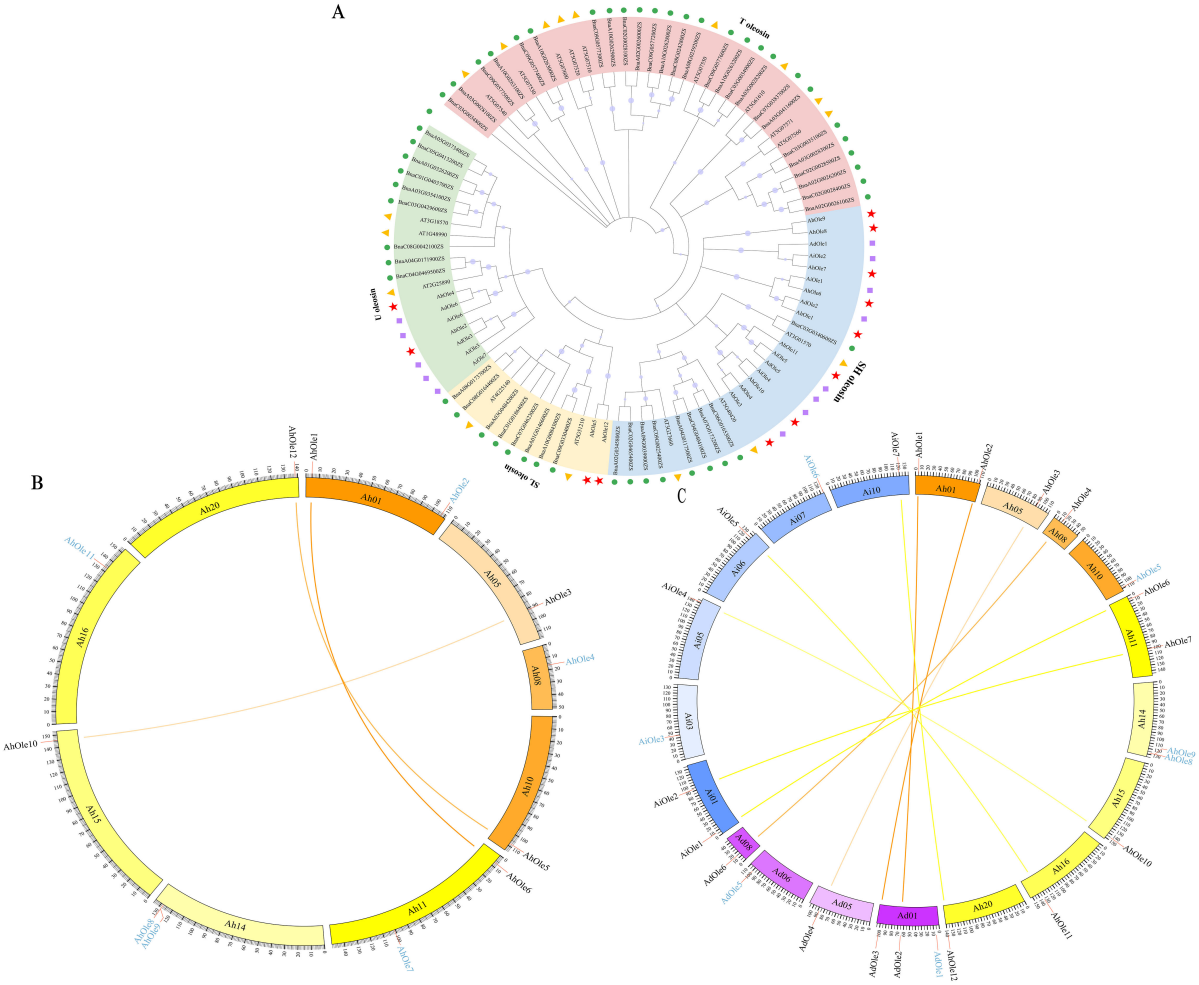
Phylogenetic and synteny analysis of oleosin genes in peanut. **(A)** Phylogenetic analysis of oleosins from *A*. *hypogaea*, *A*. *duranensis*, *A*. *ipanesis*, *A*. *thaliana* and *B. napus*. The oleosin family members were categorized into four groups: T, U, SL and SH lineages. Various shapes denoted different plant species. **(B)** Syntenic analysis of *AhOle* genes on the chromosomes in *A*. *hypogaea*. Ah01–Ah10, chromosomes from the A subgenome; Ah11–Ah20, chromosomes from the B subgenome. The colored lines indicate the syntenic gene pairs between A and B subgenomes. The blue character represents the non-syntenic AhOle genes. **(C)** Interspecies collinearity analysis in *A*. *hypogaea*, *A*. *duranensis*, and *A*. *ipaensis*. Ah01–Ah20, chromosomes from *A*. *hypogaea*; Ad01-Ad10, chromosomes from *A*. *duranensis*; Ai01-Ai10, chromosomes from *A*. *ipaensis.* The colored lines indicate the syntenic gene pairs between *A*. *hypogaea* and *A*. *duranensis*, as well as between *A*. *hypogaea* and *A*. *ipaensis*. The blue character represents the non-syntenic genes.

An un-rooted phylogenetic tree was constructed in MEGA X based on the protein sequences sourced from *A. hypogaea* (12 AhOles), *A. duranensis* (6 AdOles), *A. ipanesis* (7 AiOles), *A. thaliana* (17 AtOles) and *B. napus* (48 BnOles). The oleosin proteins were divided into four groups: U, SL, SH and T lineages. AhOle was classified into U, SL and SH lineages, but no AhOle was detected in T lineage (**Figure 3A**). AhOle2 and AhOle4 were grouped into the U lineage, while AhOle5 and AhOle12 were grouped into the SL lineage. Eight AhOles were grouped into the SH lineage, including AhOle1, AhOle3, AhOle6, AhOle7, AhOle8, AhOle9, AhOle10 and AhOle11 (**Figure 3A**). These results indicate that only three common oleosin proteins are present in peanut: U, SL and SH oleosins.

Through intragenomic comparison analysis, three collinear gene pairs in *A. hypogaea* were obtained (**Figure 3B**). AhOle genes on Chr01, Chr05 and Chr10 were syntenic to AhOle genes on corresponding Chr11, 15 and 20. The AhOle2 and AhOle4 in subgenome A were not paired, as well as four AhOle genes in subgeome B (AhOle7, AhOle8, AhOle9 and AhOle11) (**Figure 3B**).

To achieve a deeper understanding of the phylogenetic mechanisms occurring in the peanut oleosin family, the synteny maps of the oleosin genes in the *A. hypogaea* (AABB) genome and their homologous genes in two ancestral species *A. duranensis* (AA) and *A. ipaensis* (BB) were constructed. Through intergenomic comparison analysis, four gene pairs between *A. hypogaea* and *A. duranensis* and five gene pairs between *A. hypogaea* and *A. ipaensis* were identified (**Figure 3C**). The syntenic genes of AdOle1 and AdOle5 in *A. duranensis* were not found in *A. hypogaea*, as well as AiOle3 and AiOle6 in *A. ipaensis.* In addition, no syntenic genes of AhOle5, AhOle8 and AhOle9 were identified in *A. duranensis* and *A. ipaensis* (**Figure 3C**). AhOle8 and AhOle9 were found to be homologous gene pairs, potentially resulting from tandem duplication events (**Figure 3A**).

### 3.3 Expression profiles of oleosin genes in peanut

**Figure 4:**
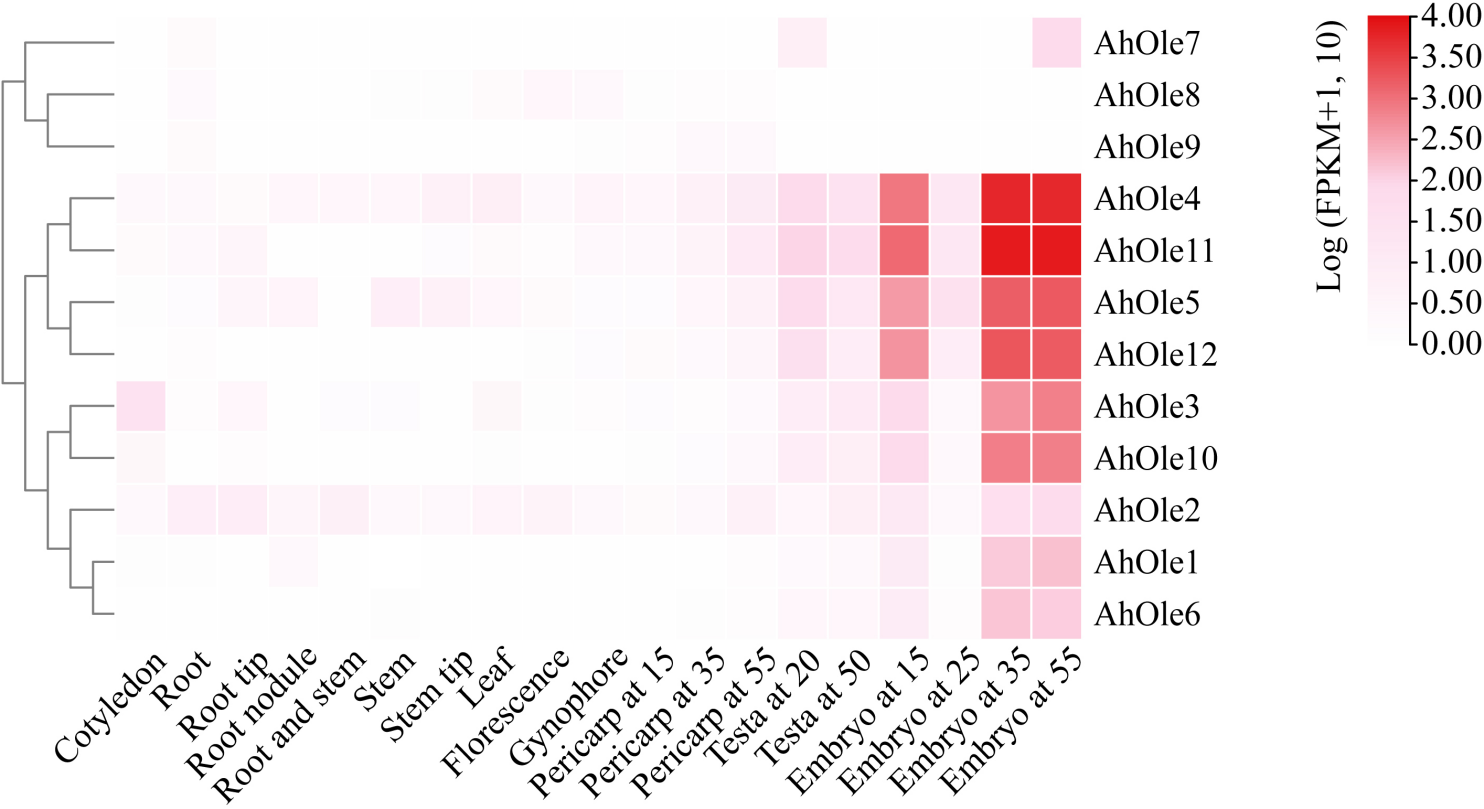
Expression profiles of *AhOle* genes across different tissues and developmental stages in peanut.

To elucidate the roles of *AhOle* genes during different growth and developmental stages in peanuts, the expression patterns of these genes were analyzed using transcriptome data from the reference *A. hypogaea* cv. ShiTouQi. A heat-map of *AhOle* genes was created to demonstrate their expression profile (**Figure 4**). *AhOle8* and *AhOle9* were not expressed in any of the tested tissues, whereas other *AhOle* genes exhibit high expression. Among them, the expression levels of *AhOle4*, *AhOle5*, *AhOle11* and *AhOle12* were found to be the highest during seed development. The expression level of *AhOle3* and *AhOle10* in developing seeds were observed moderately high, followed by *AhOle1*, *AhOle6* and *AhOle2*. *AhOle7* was exclusively expressed at the initial and final stages of seed development (**Figure 4**).

To validate the transcriptome data, the expression levels of SH (*AhOle1/6, AhOle3/10, and AhOle11*), SL (*AhOle5/12*), and U (*AhOle2*) Oleosin genes were further investigated in Zhonghua12 tissues. The results indicated that eight genes exhibited significantly different expression patterns (**Supplementary Figure S1**). *AhOle1/6* are expressed in stems, leaves, and seeds. *AhOle2* and *AhOle11* are expressed in all the tissues, and transcription abundance is the highest in seeds. *AhOle3/10* exhibited predominant expression in seeds and displayed a pattern of increasing expression level along with seed development (**Supplementary Figure S1A**). The expression of *AhOle5/12* is significantly higher in the later stage of seeds and leaves than in other tissues (**Supplementary Figure S1A**). In addition, comparison analysis of *AhOle1/6, AhOle2, AhOle3/10, AhOle5/12*, and *AhOle11* expression throughout peanut seed developmental stage showed higher transcription accumulation during 30 and 60 DAP, with *AhOle11* demonstrating the highest expression abundance among those genes (**Supplementary Figure S1B**). This result indicates that qPCR analysis of *AhOle* gene expression patterns was consistent with transcriptome data.

### 3.4 Cis-elements of *AhOles* gene and respond to stress analysis

**Figure 5:**
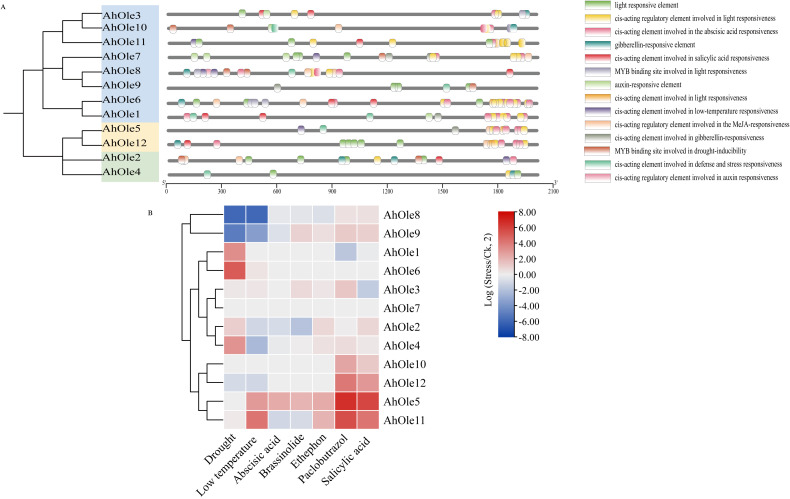
Predicted cis-elements in the promoter regions of *AhOle* genes along with their responses to abiotic stresses and plant hormones. **(A)** Predicted cis-elements in the promoter regions of *AhOle* genes. **(B)** The expression profiles of *AhOle* genes in peanut leaves treated with drought and cold stresses, as well as six plant hormones. The transcriptome data were download from Peanut Genome Resouce.

Oleosin genes have been reported to be regulated in response to various abiotic stresses. The cis-elements within promoters play a crucial role in modulating gene transcription. To investigate the potential cis-elements involved in response of peanut to abiotic stresses, the upstream 2000-bp regions of *AhOle* genes were analyzed. A diverse array of cis-element was identified, including those associated with development, phytohormone responses, and stress tolerance (**Figure 5A**). For instance, the LTR element (CCGAAA), which is linked to low temperature tolerance, was identified in promoters of *AhOle2*, *AhOle7*, *AhOle8* and *AhOle11*. The MYB binding sites (TAACTG) implicated in drought inducibility was found in all *AhOle* gene promoters. The ABRE element (AAGAA-motif), which responds to abscisic acid (ABA), was present in the promoters of *AhOle1*, *AhOle2*, *AhOle3*, *AhOle5*, *AhOle6*, *AhOle7*, *AhOle8, AhOle10*, *AhOle11* and *AhOle12*. The TGACG-motif and CGTCA-motif, both responsive to jasmonic acid (JA), were identified in the promoters of *AhOle2*, *AhOle5*, *AhOle6*, *AhOle7*, *AhOle8, AhOle10*, *AhOle11* and *AhOle12*. The TCA-element, which respond to salicylic acid (SA), was detected in the promoters of *AhOle1*, *AhOle2*, *AhOle3*, *AhOle6*, *AhOle8, AhOle11* and *AhOle12*. The GARE-motif, associated with gibberellin (GA) response, was present in the promoters of *AhOle2*, *AhOle3*, *AhOle4*, *AhOle5*, *AhOle6*, *AhOle7, AhOle10*, *AhOle11* and *AhOle12*. The ERELEE4-motif, involved in ethylene response, was found in the promoters of *AhOle4*, *AhOle5*, *AhOle6*, *AhOle8, AhOle10*, *AhOle11* and *AhOle12* (**Figure 5A**).

The transcriptome data of peanut leaves subjected to drought and cold stress, as well as those treated with plant hormone, were downloaded and analyzed (**Figure 5B**). The expression levels of *AhOle1*, *AhOle4* and *AhOle6* were significantly up-regulated under drought stress, while those of *AhOle8* and *AhOle9* were significantly down-regulated. Under cold stress, the expression level of *AhOle11* was significantly up-regulated, whereas those of *AhOle4*, *AhOle8* and *AhOle9* were significantly down-regulated. Following treatment with paclobutrazol, the expression level of *AhOle5*, *AhOle10* and *AhOle11* were significantly up-regulated. Additionally, the expression level of *AhOle5* and *AhOle11* were significantly up-regulated after treated with salicylic acid (**Figure 5B**).

The expression of *AhOle8* was down-regulated in response to both drought and cold stresses, along with three MYB elements and one LTR element in its promoter. The expression of *AhOle11* was up-regulated upon exposure to salicylic acid, with a TCA-element detected in its promoter. Meanwhile, the expression levels of *AhOle5*, *AhOle10*, and *AhOle11* were up-regulated following treatments with abscisic acid, salicylic acid, and ethephon, respectively, which is consistent with the predicted cis-elements. These findings suggested that *AhOle* genes play an important role in peanut development and responses to abiotic stress.

### 3.5 Subcellular localization of *AhOle11*

**Figure 6:**
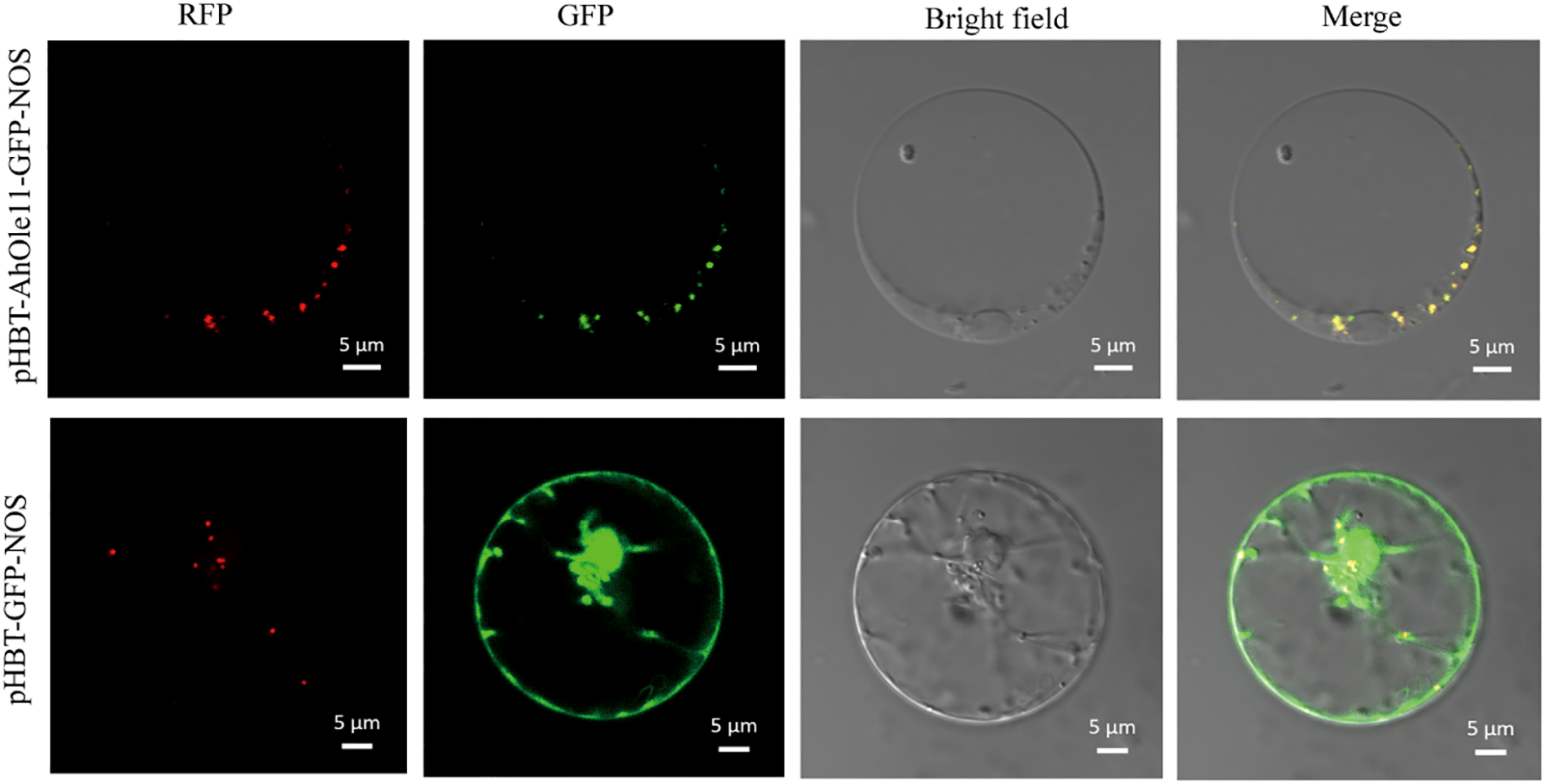
Subcellular localization of AhOle11 in *Arabidopsis* protoplast cell. pHBT-GFP-NOS was used as a control, RFP was an oil body marker, Bar= 5µm.

The *AhOle11* gene was selected for further functional analysis, as it belongs to the SH lineage and exhibited the highest expression level during seed development (**Figures 3**, **4**). To investigate the subcellular localization of AhOle11, it was fused with GFP and subsequently co-expressed with an oil body marker in *Arabidopsis* protoplasts. As anticipated, the green fluorescence from the empty vector was observed throughout the entire cell. In contrast, the green fluorescence of AhOle11 was completely co-localized with the red fluorescence of the oil body marker (**Figure 6**). These results indicated that AhOle11 was located within the oil body, where oleosin exerts its function.

### 3.6 Overexpression of *AhOle11* in *Arabidopsis*

**Figure 7:**
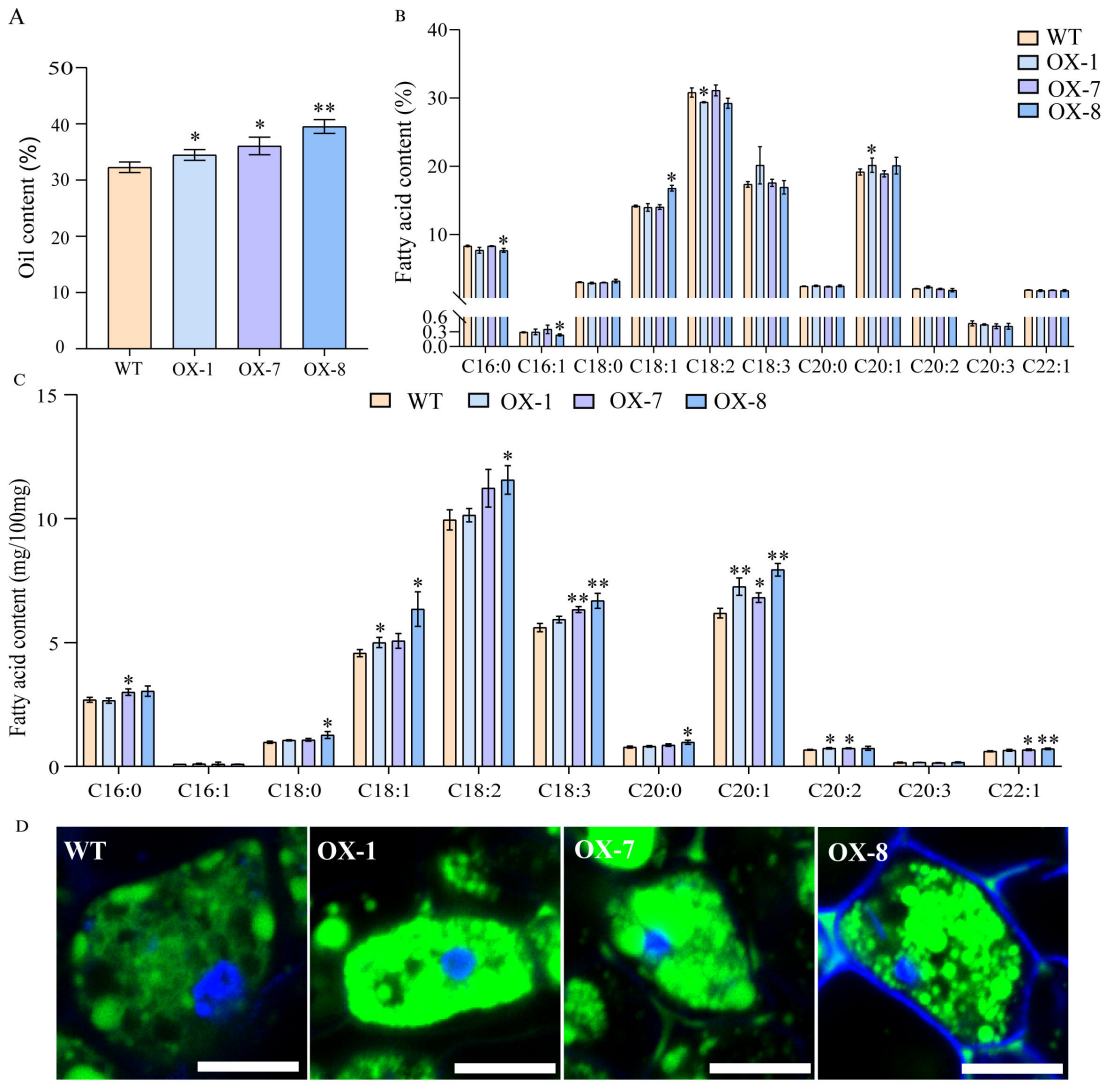
The oil contents and fatty acid compositions in *Arabidopsis* seeds harvested from wild-type and *AhOle11*-overexpressing lines. **(A)** Oil content in transgenic seeds and the wild type (WT). **(B)** Relative contents of fatty acids transgenic seeds and the WT. C16:0, palmitic acid; C16:1, palmitoleic acid; C18:0, stearic acid; C18:1, oleic acid; C18:2, linoleic acid; C18:3, linolenic acid; C20:0, arachidic acid; C20:1, eicosenoic acid; C20:2, eicosadienoic acid; C20:3, eicosatrienoic acid; C22:1, erucic acid. **(C)** Absolute contents of fatty acids transgenic seeds and the WT. **(D)** The oil body stained with bodipy. The green was oil body, Bar=10µm. The asterisks indicate significant differences between WT and Overexpression line according to Student’s t-test, * and ** mean significant correlation at the 0.05 and 0.01 probability levels, respectively.

To further confirm the function of AhOle11 in plants, the *AhOle11* gene was overexpressed in the *Arabidopsis*. A total of ten *AhOle11*-overexpressing lines were obtained. The oil content and fatty acid composition of DsRed positive seeds from three homozygous T_3_ lines (OX-1, OX-7, and OX-8) were determined by gas chromatography. The oil contents in *Arabidopsis* WT seeds varied between 31.2% and 32.9%, whereas the oil content in *AhOle11*-overexpressing lines ranged from 33.7% to 40.8% (**Figure 7A**). The oil contents in seeds of *AhOle11*-overexpressing lines were significantly higher than that in WT seeds. Among the overexpressing lines, the OX-8 line exhibited the highest oil content (38.4%~40.8%), which represents a 22.4% increase compared to the control (**Figure 7A**). Then the oil bodies in seeds of WT and overexpressing lines were analyzed. Compared to WT, a significant higher oil body density was observed in the overexpressing lines, whereas no significant difference in oil body size was observed between them (**Figure 7D**). The result visibly illustrated that *AhOle11*-overexpressing lines contained higher level of oil accumulation in seed more than WT.

In addition, the absolute quantification of fatty acid levels was examined in lines overexpressing *AhOle11*. Compared to WT, significant increases in fatty acid contents were observed in the following: C18:1, C20:1 and C20:2 in OX-1; C16:0, C18:3, C20:1, C20:2 and C22:1 in OX-7; as well as C18:0, C18:1, C18:2, C18:3, C20:0, C20:1 and C22:1 in OX-8 (**Figure 7C**). In terms of the relative fatty acid compositions, the contents of C20:1 in OX-1 and C18:1 in OX-8 were significantly elevated, while the contents of C18:2 in OX-1 and C16:0 as well as C16:1 in OX-8 were significantly reduced (**Figure 7B**). In summary, overexpression of *AhOle11* led to a significant increase in total fatty acid production, while the overall fatty acid composition remained largely consistent.

## 4 Discussion

Oleosin proteins are proposed to function as stabilizers that maintaining the integrity of oil bodies, preventing their aggregation and fusion (Tzen and Huang, 1992). They play critical roles in energy provision during seed development and germination. In this study, a total of 12 *AhOle* genes were identified in peanut, an important oil crop with global significance (**Table 1**). The oleosin gene family has been identified in *Arabidopsis*, rapeseed, cotton and sorghum, comprising 17, 48, 25 and 3 members in their respective genomes (Kim et al., 2002; Chen et al., 2019; Ojha et al., 2021; Yuan et al., 2021). Six and seven oleosin genes were identified in *A. duranensis* (AA genome) and *A. ipaensis* (BB genome), respectively, which are the ancestral species of cultivated peanut (AABB genome) (Jiang et al., 2023). The number of oleosin genes in cultivated peanut was nearly equivalent to the combined total of oleosin genes in its two ancestral peanut species. Gene duplication and gene loss were also observed in the oleosin gene family. The syntenic genes for *AdOle1* and *AdOle5* in *A. duranensis* and *AiOle3* and *AiOle6* in *A. ipaensis* were not found in cultivated peanut (**Figure 3C**)*. AdOle* genes always appear in pairs with *AiOle* genes, and nearly all of these homologous *AdOle* or *AiOle* genes maintained a syntenic relationship with *AhOle* genes (**Figure 3C**). A tandem duplication event was observed, as evidenced by the homologous gene pairs *AhOle8 and AhOle9* being located on the same chromosome (**Figure 3B**).

The common structural features of oleosin proteins, characterized by the conserved oleosin domain (PF01277), which is proposed to consist of *β*-strand structure and to interact with lipids (Huang, 1996; Umate, 2012). All the identified AhOles contained this conserved oleosin domain (**Figure 1B**), supporting that the genes identified in this study are members of the oleosin gene family. The molecular weight of AhOles ranged from 14.3 to 22.0 kDa (**Table 1**), consistent with the previously reported range of 15 to 26 kDa in other plants (Hsieh and Huang, 2004). The PI value of AhOles ranged from 4.02 to 10.08 (**Table 1**), aligning with the values observed for oleosin members in cotton, *Theaceae*, and wild peanuts (Yuan et al., 2021; Jiang et al., 2023; Zhang et al., 2023). Gene structure analysis showed that *AhOle* genes contain a single exon without any intron, which is in agreement with previously reported findings (Huang and Huang, 2015). It has been hypothesized that the oil body proteins may have acquired introns during early embryonic evolution, but subsequently lost them over time (Liu et al., 2012).

The phylogeny of the oleosin gene family in land plants has revealed that oleosin genes could be classified into six lineages: M, P, T, U, SL and SH (Huang, 2017). P oleosins have been identified in liverworts, mosses, ferns and gymnosperms, while M oleosins have been discovered exclusively in *Lauraceae* and T oleosins solely in *Brassicaceae*. U oleosins are ubiquitously present in all land plants, including *Selaginella moellendorffii*. SL oleosins are found in seeds of both gymnosperms and angiosperms, while SH oleosins predominantly found in seeds of angiosperms (Kim et al., 2002; Fang et al., 2014; Huang and Huang, 2015). In this study, the *AhOle* genes were divided into three lineages: U, SL, and SH (**Figure 3A**). No T oleosin was identified in peanut, which is specifically expressed in pollens during chorionic formation and pollen development (Kim et al., 2002). AhOle2 and AhOle4 were grouped into the U lineage, while AhOle5 and AhOle12 were grouped into the SL lineage. Eight AhOles were grouped into the SH lineage, including AhOle1, AhOle3, AhOle6, AhOle7, AhOle8, AhOle9, AhOle10 and AhOle11 (**Figure 3A**). The expression pattern of U, SL and SH oleosins in *Oryza sativa* and *Zea mays* showed that SH and SL oleosins mainly expressed in developing seeds, whereas U oleosins expressed across different tissues with lower expression level (Huang and Huang, 2015). Notably, both *AhOle2* and *AhOle4*, which belong to the U lineage, were expressed across all tissues, but *AhOle4* exhibited higher expression level (**Figure 4**). Apart from *AhOle8* and *AhOle9*, the remaining *AhOle* genes, which belong to SL and SH lineages, were predominantly expressed in developing seeds (**Figure 4**). The expression of *AhOle8* and *AhOle9*, which also belong to the SH lineage, were not detected in any of the examined tissues (**Figure 4**). The SL and SH oleosins have been reported to regulate both the size of oil bodies and the oil content in seeds (Chen et al., 2019; Yuan et al., 2021). Therefore, the SH oleosin gene *AhOle11*, which demonstrated the highest expression level during seed development, was selected for function analysis.

Previous studies have demonstrated that oleosin genes are involved in plant stress response by modulating oil body size and number in accordance with metabolic demands. For instance, in comparison to WT, both the *Arabidopsis Atole1* mutant and the *Atole1/Atole2* double mutant exhibited a substantially reduced germination rate when subjected to freezing treatment at -30°C (Shimada et al., 2008). When *SbOle1*, *SbOle2*, and *SbOle3* were expressed in *Atole1* mutant, the seed germination rate was restored to the WT level (Ojha et al., 2021). It suggests that oleosins can enhance freezing tolerance in seeds and promote seed germination. Furthermore, it is widely acknowledged that plant hormones play a critical role in regulating plant adaptation to stress. Specifically, the expression levels of oleosins were observed to increase 6~10 folds after treated with ABA, MeJA, and JA treatment in rapeseed and soybean (Hays et al., 1999; Guo et al., 2021). Hence, the expression levels of *AhOle* genes in response to plant stress and phytohormones were investigated. In this study, an analysis of the *AhOle* promoter regions revealed a diverse array of cis-elements associated with stress response, phytohormone regulation and plant development. These elements include motifs responsive to ABA (ABRE, AAGAA-motif), JA (TGACG-motif, CGTCA-motif), SA (TCA-element), low temperature (LTR, CCGAAA) and drought (MYB, TAACTG) (**Figure 5A**). Meanwhile, transcriptome analysis of peanut leaves treated with drought, low temperature and plant hormones revealed that the expression of *AhOle* genes were regulated by these conditions. For example, *AhOle5*, *AhOle10*, and *AhOle11* were significantly up-regulated in response to paclobutrazol (a GA inhibitor), SA and low-temperature treatments, while *AhOle8* and *AhOle9* were significantly down-regulated under low-temperature and drought conditions (**Figure 5B**). These findings suggest that *AhOle* genes play an important role in plant stress responses.

Oleosins have been reported to regulate the seed oil content in *Arabidopsis*, rapeseed and castor (Lu et al., 2018; Chen et al., 2019). The *GmOle1* was identified as a regulatory factor for oil content through GWAS. Overexpression of *GmOle1* resulted in a significant increase in the number of oil bodies, and led to a 10.6% increase in seed oil content (Zhang et al., 2019). Similarly, the overexpression of *oleosin* genes cloned form safflower, rapeseed and cotton in *Arabidopsis* also significantly elevated the seed oil content (Lu et al., 2018; Chen et al., 2019; Yuan et al., 2021). These reported oleosins were predominantly in the SH and SL lineages. In this study, the function of *AhOle11* was analyzed, a member of SH lineage (**Figure 3A**). Subcellular localization analysis showed that AhOle11 was specifically located within the oil body, where oleosin proteins perform their functions (**Figure 6**). Overexpression *AhOle11* in *Arabidopsis* resulted in a significant increase in seed oil content (**Figure 7A**), which aligns with the previously reported functions of SH oleosins from *Arabidopsis*, rapeseed and soybean (Chen et al., 2019; Yuan et al., 2021). Furthermore, oleosins have also been reported to modulate the fatty acid composition of seeds. In *Arabidopsis* oleosin knocking-out mutants, the C18:1 content decrease while the C20:1 content increased (Siloto et al., 2006). In *Arabidopsis* lines overexpressing *BnOle*, the C18:2 content increased while the C20:1 decreased (Chen et al., 2019). However, the changes in fatty acid composition observed in the three *AhOle11-*overexpressing lines in this study displayed distinct patterns. The contents of C20:1 in OX-1 and C18:1 in OX-8 were significantly elevated, while the contents of C18:2 in OX-1 and C16:0 as well as C16:1 in OX-8 were significantly reduced (**Figure 7B**). In addition, research has indicated that oleosins play a significant role in modulating the number and size of oil bodies. The size of oil bodies in rice *ole16/ole18* double mutant becomes significantly larger and more irregular, which is consistent with the findings observed in *Arabidopsis* mutants *ole2*, *ole3*, *ole4*, as well as the double mutants *ole1/ole2* and *ole1/ole3* (Shimada et al., 2008; Wu et al., 2010). In the present study, overexpression *AhOle11* led to a significant increase in oil body density, but without causing any substantial changes in oil body size dimensions (**Figure 7D**), a finding consistent with the effects observed for *GmOle1* (Zhang et al., 2019). In conclusion, these results demonstrated that the overexpression of *AhOle11* led to a significantly increase seed oil content and oil body density, indicating its potential to enhance oil accumulation in peanut seeds.

## 5 Conclusion

In this study, a total of 12 oleosin genes in peanut were identified in peanut genome. Base on phylogenetic analysis, these oleosin genes were divided into three lineages: U, SL, and SH. Apart from the *AhOle8* and *AhOle9* genes, the remaining *AhOle* genes were highly expressed during seed development. Most *AhOle* genes were found to be regulated by abiotic stresses and phytohormones, with corresponding cis-elements identified in their promoters. It suggested that *AhOle* genes play a critical role in peanut development and responses to abiotic stress. Furthermore, the SH oleosin gene *AhOle11* was cloned and characterized, which demonstrated the highest expression level during in seed development. Subcellular localization analysis revealed that AhOle11 was specifically localized to oil bodies. Overexpression of the *AhOle11* gene in *Arabidopsis* resulted in an increase in seed oil content and oil body density, with only minimal effects on fatty acid composition. Therefore, this study provides significant insights for future functional analyses of oleosin genes and present potential candidate genes to enhance oil content in peanut seeds.

## Data Availability

The datasets presented in this study can be found in online repositories. The names of the repository/repositories and accession number(s) can be found in the article/**Supplementary Material**.
