# Supplementary figures and images for: Genome-wide identification and functional characterization of oleosin genes in peanut (Arachis hypogaea L.)
Source: Front Plant Sci. 2025 Aug 6;16:1623513. doi: 10.3389/fpls.2025.1623513 (PMC12364949; doi:10.3389/fpls.2025.1623513)

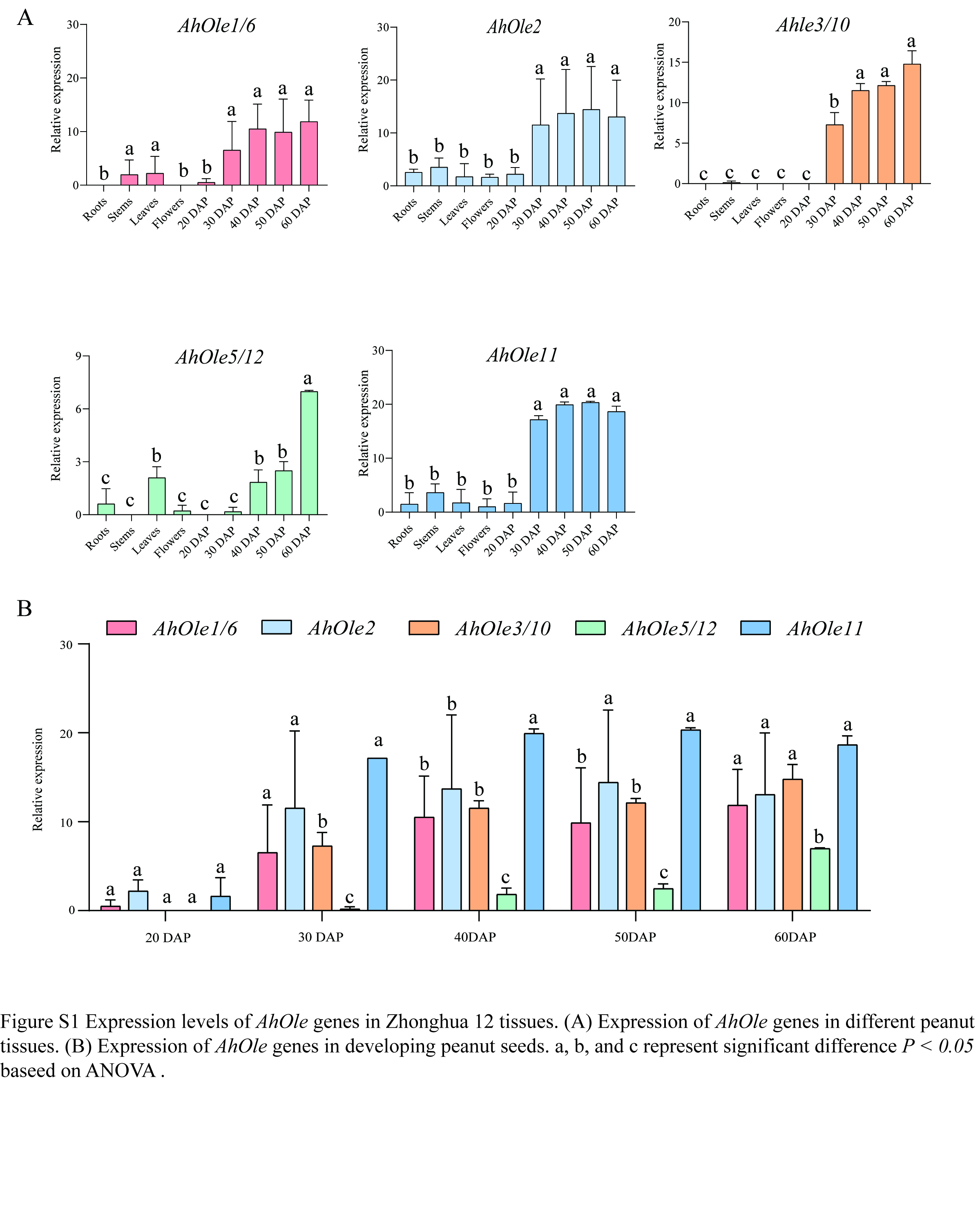

Supplement: Supplementary file 1 [file Image1.jpg]
